# Supplementary figures and images for: Lipidome profiles of postnatal day 2 vaginal swabs reflect fat composition of gilt’s postnatal diet
Source: PLoS One. 2019 Sep 26;14(9):e0215186. doi: 10.1371/journal.pone.0215186 (PMC6762109; doi:10.1371/journal.pone.0215186)

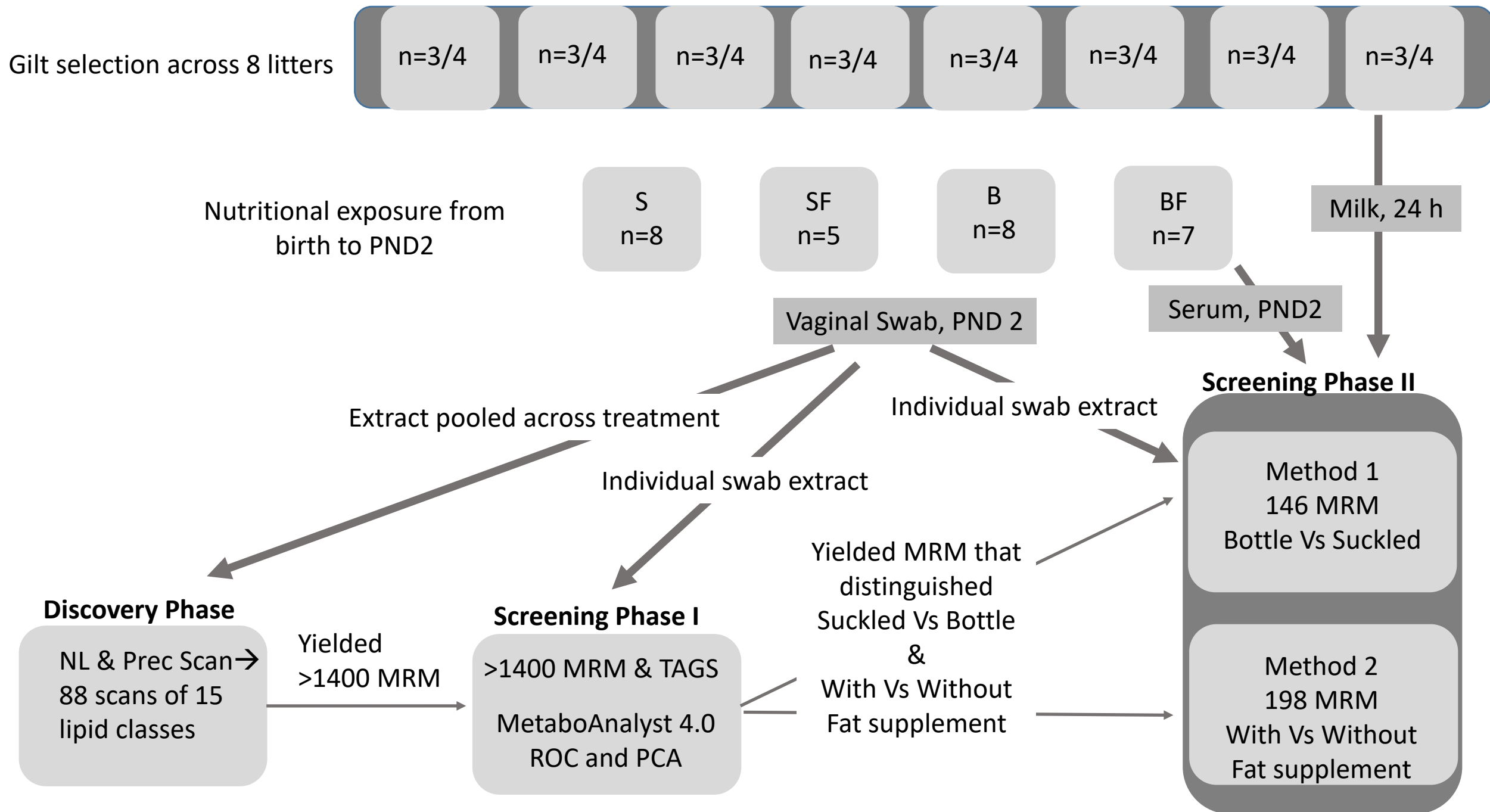

Supplement: S1 Fig — (PDF) [file pone.0215186.s001.pdf]
