# Supplementary material for: Lipidome profiles of postnatal day 2 vaginal swabs reflect fat composition of gilt’s postnatal diet
Source: PLoS One. 2019 Sep 26;14(9):e0215186. doi: 10.1371/journal.pone.0215186 (PMC6762109; doi:10.1371/journal.pone.0215186)
Supplement: S2 Fig — Comparison of relative mean intensity of TAG(46:1) in (A) nutrition source (24 h colostrum sample versus milk replacer-MR); (B) serum of suckled-S versus bottle fed milk replacer-B; and (C) vaginal swab samples in S versus B gilts. Relative mean intensity of TAG(44:0) in (E) nutrition source (24 h colostrum sample versus milk replacer-MR); (F) serum of suckled-S versus bottle fed milk replacer-B; and (G) vaginal swab samples in S versus B gilts. Relative mean intensity of PE (38:5) in (H) nutrition source (24 h colostrum sample versus milk replacer-MR); (I) serum of suckled-S versus bottle fed milk replacer-B; and (J) vaginal swab samples in S versus B gilts. Differing letters indicate statistical difference at p<0.005. (DOCX) [file pone.0215186.s002.docx]

a

a

a

b

a

b

b

b

b

b

a

a

G

H

I

F

E

D

C

B

A
